# Recolonization dynamics of the middle ear microbiota following MESNA-assisted dissection in pediatric cholesteatomatous chronic otitis media

**DOI:** 10.3389/fcimb.2026.1830192

**Published:** 2026-05-22

**Authors:** Uriel Gomez-Ramirez, Carlos De La Torre-González, Perla Villamor, Marisol Huante Guido, Araceli Contreras-Rodríguez, Norma Velázquez-Guadarrama

**Affiliations:** 1 Microbiology and Antimicrobial Resistance Research Laboratory, Hospital Infantil de México Federico Gómez, Mexico City, Mexico; 2 Laboratorio de Microbiología General, Departamento de Microbiología, Escuela Nacional de Ciencias Biológicas, Instituto Politécnico Nacional, Ciudad de México, Mexico; 3 Department of Otolaryngology, Hospital Infantil de México Federico Gómez, Mexico City, Mexico; 4 Division of Pediatric Otolaryngology, Department of Otolaryngology-Head and Neck Surgery, Stanford University, Stanford, CA, United States; 5 Laboratorio de Bacteriología Médica, Departamento de Microbiología, Escuela Nacional de Ciencias Biológicas, Instituto Politécnico Nacional, Ciudad de México, Mexico

**Keywords:** 16S rRNA sequencing, cholesteatomatous chronic otitis media (CCOM), co-occurrence networks, dysbiosis, middle ear microbiota, network analysis, pediatric otolaryngology, sodium 2-mercaptoethanesulphonate (MESNA)

## Abstract

**Introduction:**

Cholesteatomatous chronic otitis media (CCOM) remains a clinical challenge due to its high recurrence rates despite surgical intervention. Sodium 2-mercaptoethanesulphonate (MESNA) is used to assist dissection, yet its impact on the middle ear microbiome and ecological recovery remains poorly understood. The aim of this study is to characterize the microbiota of paediatric CCOM and evaluate the ecological shifts induced by MESNA-assisted surgery.

**Methods:**

We analyzed 16S rRNA gene sequences (V3-V4) from middle ear tissue of paediatric patients with CCOM (CCOM Before MESNA, n = 13; CCOM After MESNA, n = 13) and healthy controls (n = 11). Bioinformatic processing was performed via QIIME2 and DADA2. We employed a Compositional Data Analysis (CoDA) framework, centering on Aitchison distances, ALDEx2 for differential abundance, and consensus co-occurrence networks (SparCC, SPIEC-EASI, and CLR-Pearson). Functional potential was inferred using PICRUSt2.

**Results:**

CCOM was associated with a marked reduction in microbial network connectivity, decreasing from 185 edges in healthy controls to only two total edges in the CCOM Before MESNA stage. *Cutibacterium* emerged as a candidate keystone pathobiont, exhibiting profound ecological isolation and predicted metabolic shifts toward lipid catabolism and biofilm formation in dysbiotic states. MESNA application disrupted the disease-associated community equilibrium, initiating secondary succession. However, post-treatment recovery was marked by taxonomic homogenization and the expansion of *Pseudomonas* in several patients.

**Discussion:**

Our findings identify network fragmentation and functional dysbiosis as the ecological signatures of pediatric CCOM. While MESNA disrupts the dysbiotic equilibrium, it does not fully restore a healthy stable climax community within the studied timeframe, as defined in ecological succession theory. These results support a paradigm shift from simple pathogen eradication toward ecological restoration as a strategy to prevent disease recurrence in CCOM patients.

## 1 Introduction

The respiratory tract is exposed to several environmental conditions, allowing selective colonization by specific microbial communities that play a critical role in maintaining homeostasis and regulating exogenous microbial populations (Santacroce et al., 2020). In dysbiosis, the upper respiratory airways are significantly affected, contributing to infectious processes in pediatric populations. Additionally, the role of dysbiotic respiratory bacterial and viral pathogens in the increase of susceptibility to diseases (Bai et al., 2022) and ability to induce chronic-recurrent infections, such as cholesteatomatous chronic otitis media (Mittal et al., 2015; Kennedy and Singh, 2024) has been widely documented.

Cholesteatomatous chronic otitis media is characterized by the overgrowth of keratinized skin tissue in middle ear, with current accumulation in a cyst structure, known as cholesteatoma. Cholesteatoma also develops within the temporal bone, contributing to progressive bone resorption (Sarolli et al., 2021; Pachpande and Singh, 2022). Although classified as a benign tumor; cholesteatoma behaves invasively. Chronic cyst formation damages the middle ear structures, resulting in permanent hearing loss, and formation of intracranial abscesses (Pachpande and Singh, 2022). While the cause of the disease is considered as multifactorial, chronic middle ear infections have been described as the main etiology for chronic-progressive development of the cholesteatoma (Tos, 1988).

This disease is not a mortality public health problem, but rather a morbidity issue. Although its incidence is reported in 3–12 cases per 100,000 inhabitants, this condition is classified as a chronic-recurrent disease. Cholesteatoma is typically removed through mastoidectomy and ear endoscopic surgical techniques, which report high success rates due to their effectiveness; however, recurrence rates ranging from 5% - 50% are still reported (Kennedy and Singh, 2024), mainly due to inadequately removed keratinized tissue residues during mechanical dissection of the matrix, hindering disease treatment. Additional reports have described deterioration of quality of life in pediatric patients, primarily due to the requirement for lifelong care for proper humidification and cleanse of the organ, performed by qualified personnel. Several risks have been also observed as consequences of the recurrence of cholesteatoma, including the establishment and colonization by opportunistic pathogenic bacteria, fungi and virus (Blanco et al., 2014) which can be intimately associated with the chronic inflammation, and the infectious process.

To diminish recurrence events, surgeons have resorted to the chemically assisted dissection through the application of sodium 2-mercaptoethanesulphonate (MESNA) (Vincenti et al., 2014). This synthetic sulfur-containing compound hydrolyses the disulfide bonds of the polypeptide chains present in mucosal tissue, facilitating the complete removal of the cholesteatomatous matrix (de la Torre and Villamor, 2019); however, the effects of MESNA in the human middle ear microbiota have not been fully elucidated yet.

While metagenomic analyses targeting double-stranded DNA has historically dominated microbial community profiling, an emerging body of work has demonstrated the value of targeting the metabolically active microbial fraction through rRNA-derived cDNA sequencing, an approach that captures community members actively engaged in cellular processes at the moment of sampling, rather than the total microbial census including dormant or dead cells (Blazewicz et al., 2013; Feehery et al., 2013; Marotz et al., 2018). This distinction is particularly relevant in clinical low-biomass settings where the metabolically active community may differ substantially from the total community, and where host genomic background can overwhelm DNA-based approaches. Therefore, the aim of this study was to characterize the microbiota of pediatric patients with cholesteatomatous chronic otitis media, before and after the application of MESNA.

## 2 Methods

### 2.1 Specimen collection and experimental design

A total of thirty-seven middle ear tissue samples were collected for this study. The study design included two distinct cohorts: a group of independent subjects serving as Healthy Controls (HC; n = 11), and a group of pediatric patients diagnosed with cholesteatomatosus chronic otitis media (CCOM). For the CCOM group, a paired-sampling approach was employed, where biopsies were collected from the same individual both before (CCOM Before MESNA; n = 13) and after (CCOM After MESNA; n = 13) the application of 4% MESNA per 10 min to minimize inter-individual biological noise and directly evaluate the chemical effect on the local microbiota.

### 2.2 Isolation and purification of RNA, cDNA synthesis and high-throughput sequencing

All specimens were collected in 800 μL TRIzol reagent for isolation and purification of total RNA. All samples yielded RNA concentrations of 200–300 ng/µL in 30 µL elution volume, with A_260_/A_280_ ratios of approximately 1.8 - 1.9. Double-stranded DNA was then removed for synthesis of complementary DNA (cDNA). A uniform input of 1 µg total RNA per reaction was used for cDNA synthesis with random hexamers (SCRIPT cDNA Synthesis Kit, Jena Bioscience), ensuring comparable cDNA inputs across all samples. All cDNA libraries were shipped to Zymo Research (Irvine, CA, USA) for downstream PCR amplification of the bacterial 16S rRNA V3-V4 hypervariable regions, metagenomic library preparation, and Illumina MiSeq next-generation sequencing service, under standardized conditions. Negative extraction controls processed in parallel with all samples yielded no detectable amplification, confirming the absence of reagent-associated contamination.

Specimen collection in TRIzol and total RNA extraction was selected over direct DNA extraction for two methodological reasons. First, middle ear tissue biopsies yielded low biomass, and RNA-based workflows have demonstrated superior sensitivity for detecting metabolically active microbial communities in low-biomass clinical samples compared to DNA-based approaches (Feehery et al., 2013; Marotz et al., 2018). Second, given that cholesteatoma tissue contains abundant host-derived keratinized cells, RNA extraction followed by DNase treatment minimized host genomic DNA interference prior to amplification.

Random primers were used for cDNA synthesis to ensure unbiased conversion of all RNA species present, including ribosomal RNA (rRNA), messenger RNA (mRNA), and transfer RNA (tRNA). In total RNA preparations from microbial communities, rRNA typically constitutes 80–90% of the total RNA mass (Feehery et al., 2013), meaning that the cDNA pool is predominantly rRNA-derived. Subsequent PCR amplification with 16S rRNA-specific V3-V4 primers therefore selectively amplifies rRNA-derived cDNA sequences, functionally analogous to standard 16S amplicon sequencing of genomic DNA at the community composition level.

It is acknowledged, however, that rRNA-derived cDNA templates may preferentially represent metabolically active or rapidly growing community members, as rRNA copy number correlates with cellular growth rate (Blazewicz et al., 2013). This introduces a modest but relevant bias relative to DNA-based amplicon sequencing, which detects all community members regardless of metabolic state. Consequently, our compositional profiles should be interpreted as reflecting the active microbial community rather than total community membership. Importantly, all downstream bioinformatic analyses—including PICRUSt2 functional inference—were conducted following validated 16S amplicon pipelines. While PICRUSt2 was originally developed and benchmarked for DNA-derived amplicons, its application to rRNA-derived cDNA amplicons has been reported in the literature under the assumption that community composition, rather than absolute biomass, drives functional predictions (Jovel et al., 2016). Results should therefore be interpreted as reflecting predicted metabolic potential of the active community, and metatranscriptomic or metaproteomic validation is required to confirm actual pathway activity.

### 2.3 Bioinformatics and compositional data framework

Raw sequences were imported into the Quantitative Insights Into Microbial Ecology 2 (QIIME2) v. amplicon-2024.10 (Bolyen et al., 2019) for processing. Sequence quality control, denoising and chimera removal were performed using the DADA2 algorithm (Callahan et al., 2016) to infer amplicon sequence variants (ASVs). Taxonomic assignment was conducted using the SILVA 138.1 (Quast et al., 2013) database as reference, with sequences classified at 97% sequence identity threshold. Diversity analyses were conducted as well.

It should be noted that while CCOM Before and After MESNA samples were collected from the same individuals using a paired design, the Healthy Control cohort comprised independent subjects recruited cross-sectionally. This structural heterogeneity was not explicitly modelled in the statistical framework; all three groups were treated as independent observations in diversity analyses and PERMANOVA, consistent with standard practice in exploratory microbiome studies. A full discussion of the implications of this design, including recommended analytical approaches for future studies, is provided in Section 4.9.

To address the compositional nature of microbiomic data, downstream analyses were conducted in R (v4.4.3) using a Compositional Data Analysis (CoDA) framework (Gloor et al., 2017). Alpha diversity indices, including Chao1 richness (Chao, 1984), Shannon entropy (Shannon, 1948), and Simpson’s index (Simpson, 1949), were calculated to evaluate microbial diversity within samples. Statistical significance between study groups was determined using the non-parametric Wilcoxon rank-sum test. To account for multiple comparisons and minimize the false discovery rate, *p*-values were adjusted using the Benjamini-Hochberg FDR correction (Benjamini and Hochberg, 1995). All statistical analyses and visualizations were conducted in the R environment (R Core Team, 2023) using the tidyverse, ggpubr, and rstatix packages.

For Beta diversity, a Centered Log-Ratio (CLR) transformation (Anderson, 2017) was applied to raw counts to calculate Aitchison distances (Gloor et al., 2017), and global community shifts were evaluated via PERMANOVA with 9,999 permutations (Anderson, 2017).

Differential abundance was determined using the ALDEx2 (v1.36.0) algorithm (Fernandes et al., 2014), modeling technical variation through 128 Monte Carlo instances drawn from a Dirichlet distribution. Significance was defined by a FDR-adjusted *q* < 0.05 (Benjamini-Hochberg FDR correction) and a biological effect size |*Effect*| > 0.2. To identify robust predictive signatures, a Random Forest classifier (500 trees, *randomForest* v4.7) (Breiman, 2001) was trained on genus-level relative abundances to discriminate among clinical groups. Prior to model training, features with near-zero variance were removed using the *caret* package. Feature selection was performed using the Boruta algorithm (*Boruta* v8.0, maxRuns = 100) (Kursa and Rudnicki, 2010), retaining only confirmed informative genera. Model performance was assessed by five-fold cross-validation and out-of-bag error estimation. Multiclass discrimination was quantified using the multiclass AUC statistic (pROC package) (Robin et al., 2011).

Microbial co-occurrence networks were reconstructed using a consensus approach to minimize spurious correlations, intersecting three independent algorithms: SparCC (Friedman and Alm, 2012), SPIEC-EASI (Kurtz et al., 2015), and CLR-Pearson correlations (Aitchison, 1982).

Functional potential was predicted using PICRUSt2 (v2.5.3) (Douglas et al., 2020) to infer KEGG Orthologs (KOs), Enzyme Commission (EC) numbers, and MetaCyc metabolic pathways from ASV sequences. As an exploratory complement to the primary compositional framework, taxonomic and functional biomarkers were visualized using the Linear Discriminant Analysis Effect Size (LEfSe) algorithm (Segata et al., 2011), applying a Kruskal-Wallis test (α = 0.05) and an LDA score threshold > 2.0. LEfSe was employed for hypothesis generation and visualization rather than as a primary inferential method; all formal differential abundance inference relied on ALDEx2.

## 3 Results

### 3.1 Alpha diversity metrics reveal diversity collapse signatures in middle ear dysbiosis

**Figure 1:**
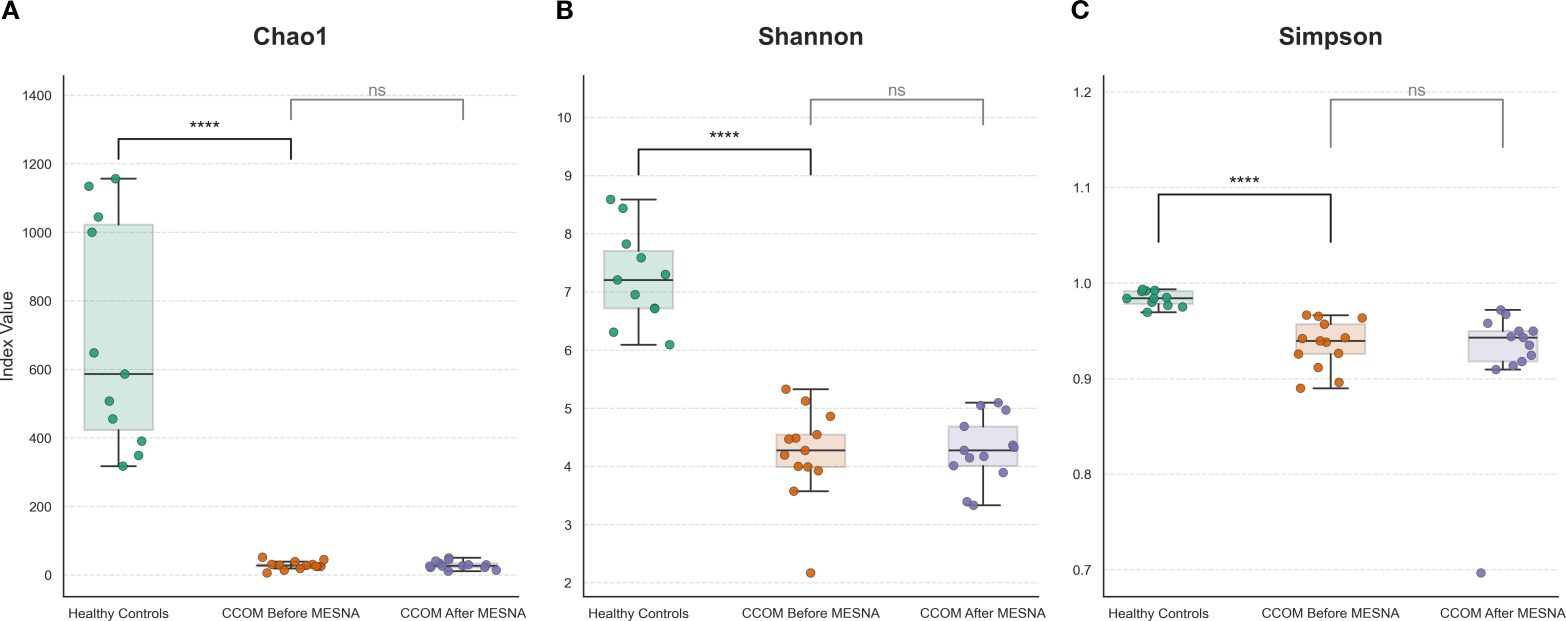
Alpha diversity metrics across study groups. **(A)** Shannon’s diversity index quantifying both richness and evenness. **(B)** Chao1 richness estimator indicating total species diversity. **(C)** Simpson’s diversity index measuring dominance and evenness. Healthy controls (HC, n = 11) show significantly higher diversity across all metrics. Statistical comparisons performed using Wilcoxon rank-sum test with Benjamini-Hochberg FDR correction. **** *p* < 0.0001. ns, non-significant (*p* > 0.05). Box plots show median (center line), interquartile range (box), and individual data points (dots).

Significant differences in alpha diversity (**Figure 1**) were observed when comparing the Healthy Control group against CCOM patients. For all three metrics evaluated—Chao1, Shannon, and Simpson—the Healthy Control group exhibited substantially higher diversity levels compared to both CCOM stages. Specifically, the Chao1 richness index showed a drastic reduction in the CCOM Before MESNA group compared to Controls (adjusted *p* < 0.0001). Similarly, Shannon and Simpson indices revealed a significant loss of evenness and diversity in patients before treatment (adjusted *p* < 0.0001).

In contrast, no significant differences were observed in any alpha diversity metric when comparing CCOM patients before and after the application of MESNA (Chao1: *p* = 0.81; Shannon: *p* = 0.54; Simpson: *p* = 0.42), indicating that the diversity remained stable and significantly lower than the healthy baseline throughout these stages.

### 3.2 Genus-level architecture across clinical states

**Figure 2:**
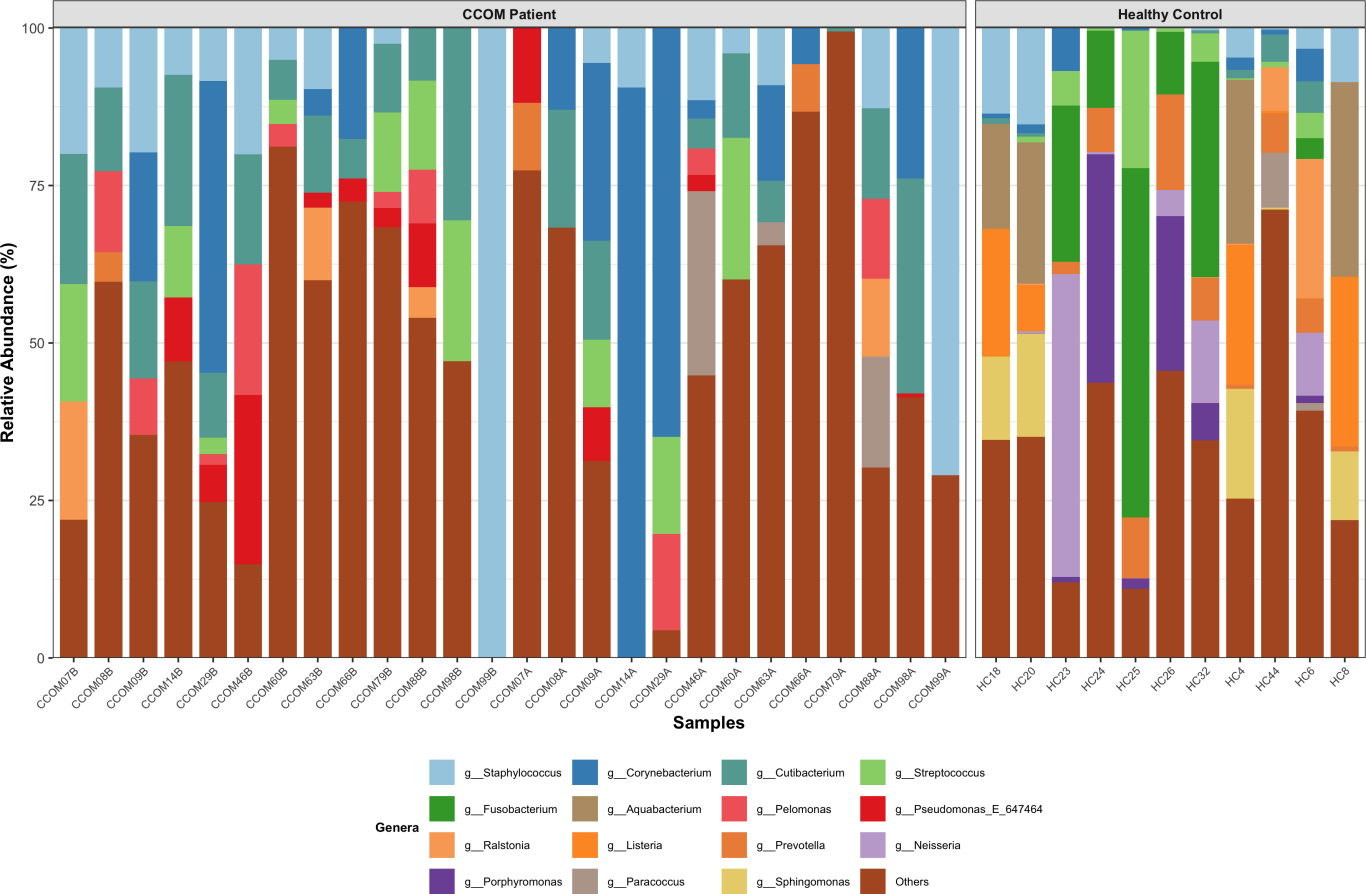
Taxonomic composition at genus level across study groups. Stacked bar plots showing relative abundances of bacterial genera in healthy controls (HC), CCOM Before MESNA **(B)**, and CCOM after MESNA **(A)**. Each bar represents and individual patient. Genera with relative abundances < 1% are grouped as “Others”. Dominance of *Fusobacterium* and *Aquabacterium* in HC, *Staphylococcus* and *Cutibacterium* in CCOM before MESNA and reshuffling with *Corynebacterium* dominance and *Pseudomonas* emergence in CCOM after MESNA samples was observed.

The metagenomic profile of the Healthy Control (HC, **Figure 2**) cohort was characterized by a diverse assemblage of commensal taxa, although no single genus was present in 100% of the individuals, highlighting the natural inter-individual variability of the middle ear microbiota. The community was ecologically dominated by *Fusobacterium*, which exhibited the highest average abundance (up to 55.45%), followed by *Aquabacterium* (up to 30.88%) and *Listeria* (up to 26.93%). Notably, *Prevotella* stood out as the most ubiquitous genus, being identified in 81.8% of the subjects with a consistent but moderate presence (up to 15.15%). In contrast, the “rare biosphere” of this group was represented by low-abundance taxa such as *Legionella* (up to 0.79%) and *Anaerococcus* (up to 1.08%), which contributed to the overall taxonomic richness without dominating the biomass.

In the CCOM Before MESNA group, the microbial architecture underwent a significant simplification toward a dysbiotic profile where pathobionts became highly prevalent. *Cutibacterium* emerged as a near-core pillar of this pathology, being present in 92.3% of the patients with substantial representative levels (up to 30.53%). The taxonomic dominance, however, was led by *Staphylococcus*, which reached an average abundance of 15.56%, displaying a massive expansion in specific cases (up to 100%). Other prominent members included *Corynebacterium* (up to 46.32%) and *Streptococcus* (up to 22.39%), which together established a robust but pathological microbial foundation. Minor components such as *Lawsonella* (up to 5.72%) were consistently detected, representing specialized niches within the cholesteatoma tissue before any chemical intervention.

Following the surgical application of MESNA, the metagenomic profile exhibited a dynamic taxonomic reshuffling. *Corynebacterium* became the most dominant genus in this group, significantly increasing its average proportion and showing a wide distribution across patients (up to 90.54%). While the primary drivers of the initial infection, *Staphylococcus* and *Cutibacterium*, remained present in over half of the cohort (53.8% and 61.5% prevalence, respectively), their mean abundances were attenuated to 9.49% (range up to 71%) and 8.32% (up to 34.11%). Interestingly, this group saw the emergence of taxa such as *Eubacterium*_B, which, although rare in other contexts, reached high peaks in specific post-treatment samples (mean 4.27%; range up to 55.51%). Very rare taxa, such as *Oribacterium* (mean 0.06%; range up to 0.75%), persisted at the detection limit, indicating a highly fragmented and transitioning ecological state. The remaining taxa with relative abundances below 1% were grouped into the “Others” category to simplify the ecological interpretation.

### 3.3 Aitchison distance reveals MESNA-induced individualized ecological transitions

**Figure 3:**
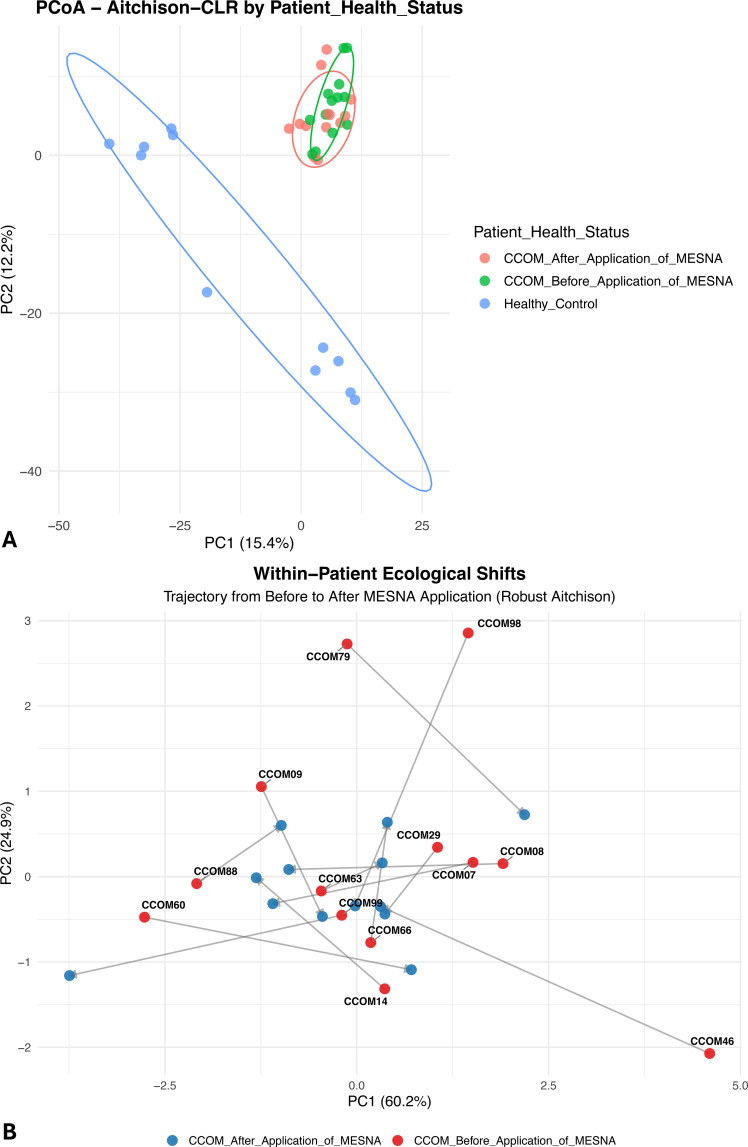
Beta diversity principal coordinate analysis (PCoA) based on Aitchison distance. **(A)** the first principal component (PC1, 15.4% variance) clearly segregates healthy controls (blue) from clinical samples. CCOM before MESNA (green) samples cluster tightly in a region defined by the *Staphylococcus*-*Cutibacterium* compositional axis, while CCOM after MESNA samples exhibit a divergent trajectory away from this pathological attractor state (Scheffer et al., 2001). PERMANOVA confirmed statistically significant separation between groups (*p* < 0.001; 9,999 permutations). Ellipses represent 95% confidence intervals. **(B)** within-patient compositional trajectories in robust Aitchison PCoA space. Arrows connect the pre-treatment (CCOM before MESNA) and post-treatment (CCOM after MESNA) coordinates of each individual patient. Patient identifiers are shown at the pre-treatment baseline. The multi-directional nature of individual trajectories confirms that MESNA-induced ecological displacement is highly stochastic and patient-specific (adonis2: F = 0.2935, R² = 0.032, *p* = 0.404, 9,999 permutations, strata = patient identity).

Compositional differences in community structure (**Figure 3**) were assessed through Principal Coordinates Analysis (PCoA) based on Aitchison distance (**Figure 3A**). The ordination demonstrated a robust spatial segregation, with the first principal component (PC1) accounting for 15.4% of the total variance. Healthy Control samples formed a highly cohesive cluster that was clearly isolated from the clinical samples along the primary axis of variation, a separation confirmed to be statistically significant by PERMANOVA (*p* < 0.001). Within the cholesteatoma cohort, a distinct shift in community centroids was observed before and after application of MESNA.

While samples in the CCOM Before MESNA group were tightly associated with a specific region of the log-ratio space defined by the *Staphylococcus*-*Cutibacterium* axis, the CCOM After MESNA group exhibited a divergent ecological trajectory.

To evaluate global compositional shifts induced by the surgical intervention, we applied a paired statistical framework using Robust Aitchison distances. A paired PERMANOVA, utilizing patient identity as a blocking factor (strata), revealed that the overall community composition did not systematically diverge into a predictable ‘post-treatment’ state across the cohort (adonis2: F = 0.2935, R^2^ = 0.032, *p* = 0.404, 9,999 permutations, strata = patient identity).

This lack of a uniform compositional shift was visually corroborated by mapping within-patient trajectories in the Aitchison PCoA space (**Figure 3B**). Notably, the primary axes captured a substantial proportion of the variance (PC1: 60.2%, PC2: 24.9%). However, individual patient trajectories exhibited highly divergent, multi-directional shifts away from their pre-treatment baselines. This visual evidence confirms that while MESNA disrupts the pre-existing dysbiotic community, the resulting ecological displacement is highly stochastic and patient-specific.

### 3.4 Taxonomic biomarkers distinguish homeostasis from transitional states

**Figure 4:**
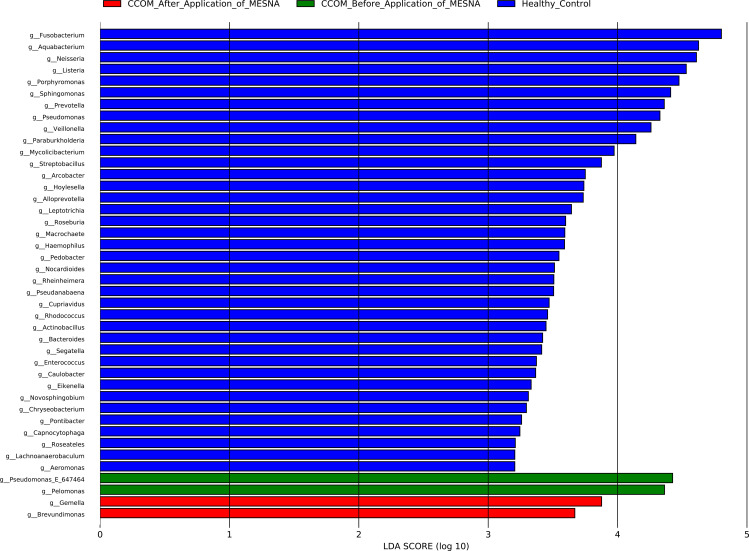
Linear discriminant analysis effect size (LEfSe) of significantly enriched genera. Healthy controls exhibit 42 discriminative genera, reflecting high taxonomic diversity. CCOM before and after MESNA shows two enriched genera, respectively. Kruskal-Wallis test (α = 0.05).

Exploratory LEfSe analysis at the genus level identified candidate taxonomic signatures across the three study groups (**Figure 4**). Healthy controls exhibited the highest number of discriminative genera (n = 42), including *Fusobacterium*, *Aquabacterium*, *Neisseria*, *Listeria*, *Prevotella*, *Veillonella*, and *Haemophilus* among others, reflecting the high taxonomic diversity.

In contrast, CCOM Before MESNA samples showed only two significantly enriched genera: *Pseudomonas* and *Pelomonas*, while CCOM After MESNA samples exhibited *Gemella* and *Brevundimonas* as discriminative genera.

### 3.5 Co-occurrence architectures reveal network fragmentation

**Figure 5:**
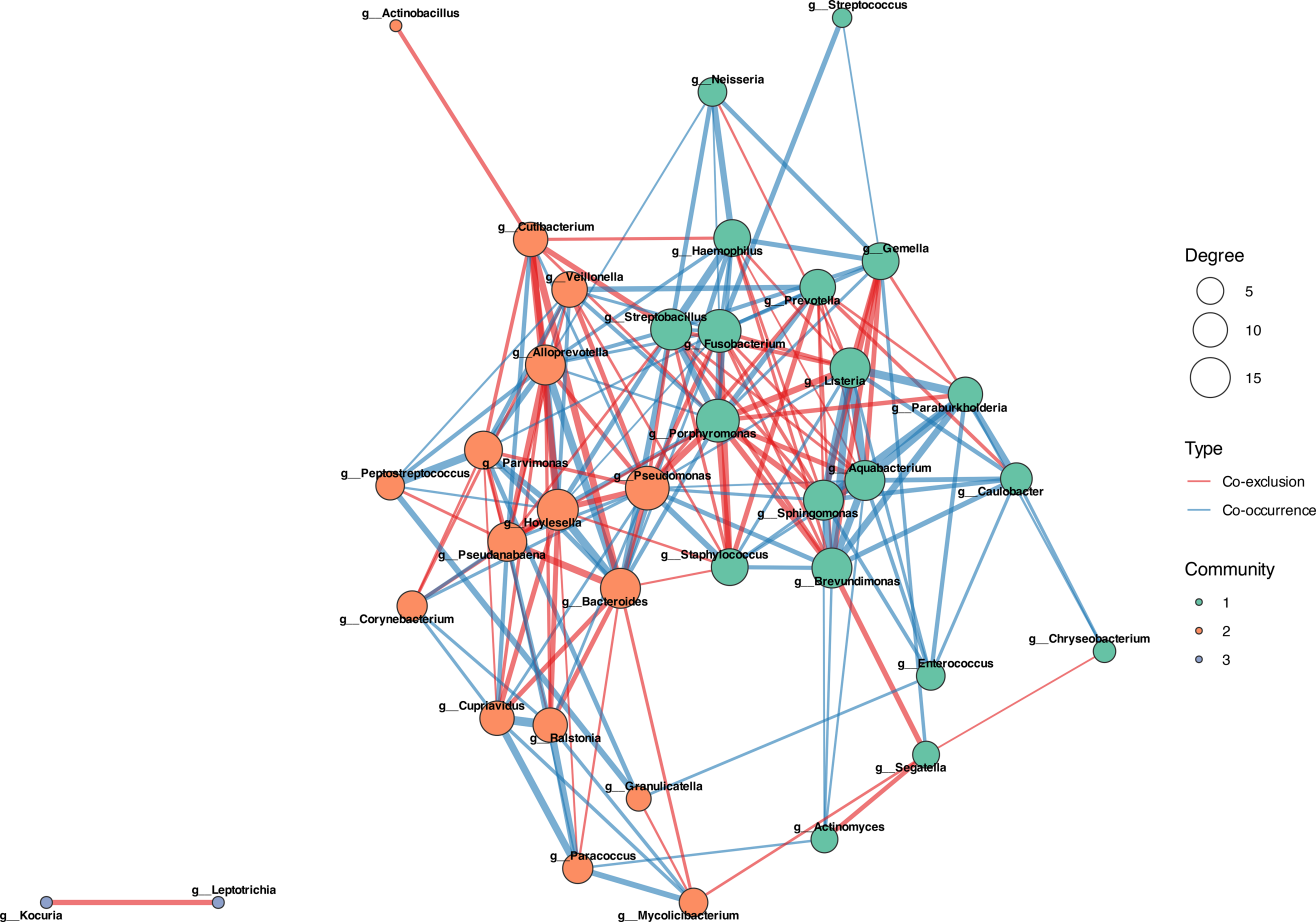
Microbial co-occurrence network in healthy controls. Network comprising 38 bacterial genera organized into three ecological communities (colored modules), with 185 total edges. Node size represents relative abundance; edge thickness indicates association strength. Blue edges: positive co-occurrence (cooperation); red edges: negative association (competitive exclusion). Key hub taxa include *Fusobacterium*, *Prevotella*, *Aquabacterium*, *Streptococcus*, and *Cutibacterium*. Network inferred using consensus approach (SparCC, SPIEC-EASI, CLR-Pearson).

Network analysis based on centered log-ratio-transformed abundance data identified distinct co-occurrence patterns across the three study groups. The healthy control microbiota exhibited a highly interconnected network structure (**Figure 5**). The network comprised 38 bacterial genera organized into three distinct ecological communities. The predominant interaction pattern was co-exclusion (negative associations), characteristic of stable, competitive communities in homeostatic equilibrium. Key hub taxa included *Fusobacterium*, *Prevotella*, *Aquabacterium*, *Streptococcus*, and *Cutibacterium*, each displaying high node degree (5–15 connections).

Co-occurrence relationships (positive associations) were primarily observed within functional guilds—among anaerobic taxa (*Fusobacterium*, *Prevotella*, *Veillonella*) and aerobic commensals (*Neisseria*, *Corynebacterium*, *Staphylococcus*, *Cutibacterium*). Notably, *Cutibacterium* participates actively in the healthy network.

**Figure 6:**
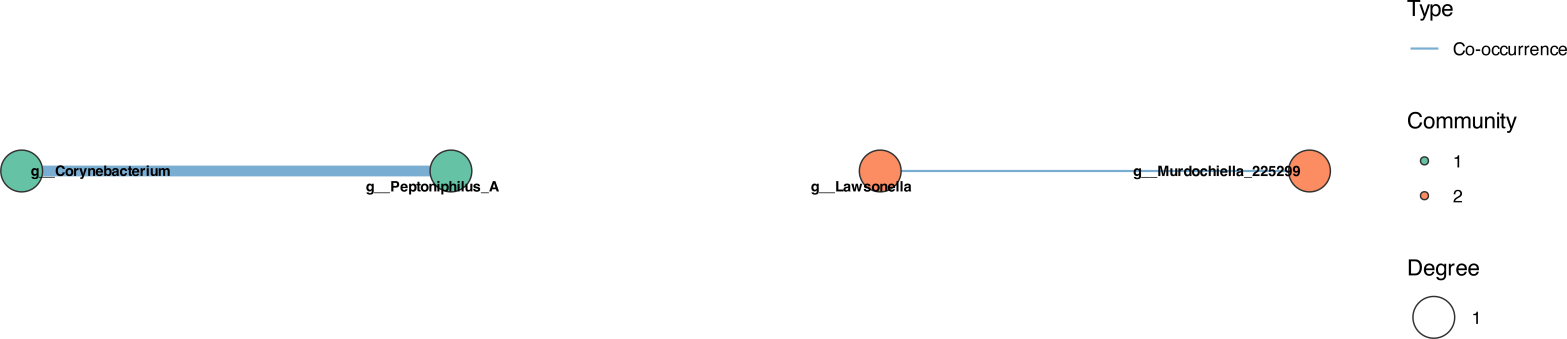
Microbial co-occurrence network before MESNA application. Network exhibits severe ecological simplification with only 4 interacting genera forming 2 isolated communities (2 total edges). Community 1: *Corynebacterium*–*Peptoniphilus*_A positive association. Community 2: *Lawsonella*–*Murdochiella* positive association. Complete absence of competitive exclusion interactions and low connectivity indicate loss of ecological resilience and regulatory mechanisms. Compared with 185 edges in healthy controls observed in **Figure 5**.

In contrast, the CCOM Before MESNA network exhibited severe ecological simplification (**Figure 6**). The network collapsed to four interacting genera organized into two isolated communities. *Corynebacterium* formed a single positive association with *Peptoniphilus_A*, while *Lawsonella* co-occurred with *Murdochiella*, forming a separate dyadic interaction.

**Figure 7:**
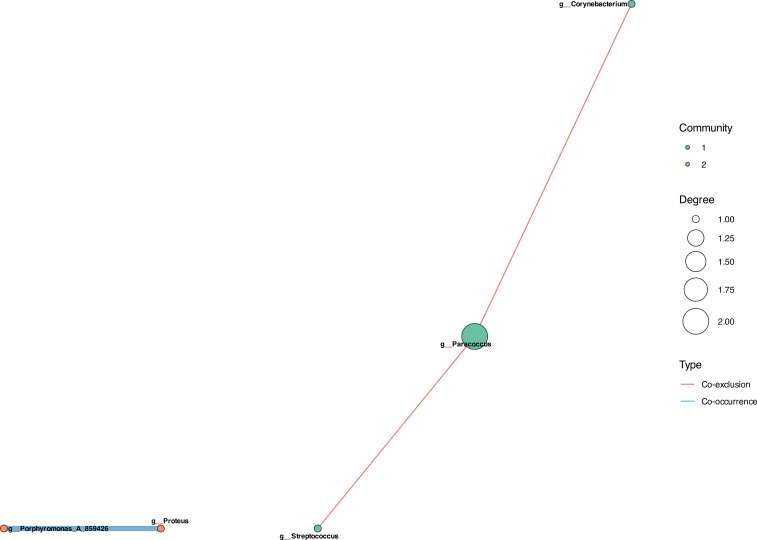
Microbial co-occurrence network after MESNA application. Network shows minimal recovery with five genera across two communities (three total edges). Community 1: *Corynebacterium*, *Paracoccus*, and *Streptococcus*. Community 2: *Porphyromonas*_A and *Proteus*. Critical distinction from CCOM before MESNA: reemergence of competitive exclusion (red edges) alongside cooperative interactions (blue edges), indicating partial restoration of ecological regulation. However, connectivity remains dramatically reduced compared to healthy state (185 edges).

Following MESNA application, the network structure remained minimal but exhibited a shift toward mixed ecological dynamics (**Figure 7**). The post-treatment network comprised five genera across two communities, featuring *both* co-occurrence and co-exclusion interactions—a critical distinction from the purely cooperative CCOM Before MESNA network.

Community 1 included *Corynebacterium*, *Paracoccus*, and *Streptococcus*, while Community 2 consisted of *Porphyromonas*_A and *Proteus*. Within Community 1, *Paracoccus* and *Streptococcus* formed a positive co-occurrence relationship, suggesting a mutualistic partnership potentially involving metabolic cross-feeding or co-tolerance to the post-surgical microenvironment. In contrast, competitive exclusion was observed between *Porphyromonas_A* and *Proteus* (inter-community), and between *Corynebacterium* and the *Paracoccus*-*Streptococcus* dyad (intra-community).

### 3.6 Predicted functional shifts from biosynthesis to resource competition

Exploratory functional inference using PICRUSt2 and LEfSe identified potential metabolic pathways based on reference genomes, which should be considered hypothesis-generating rather than direct measurements of enzymatic activity.

**Figure 8:**
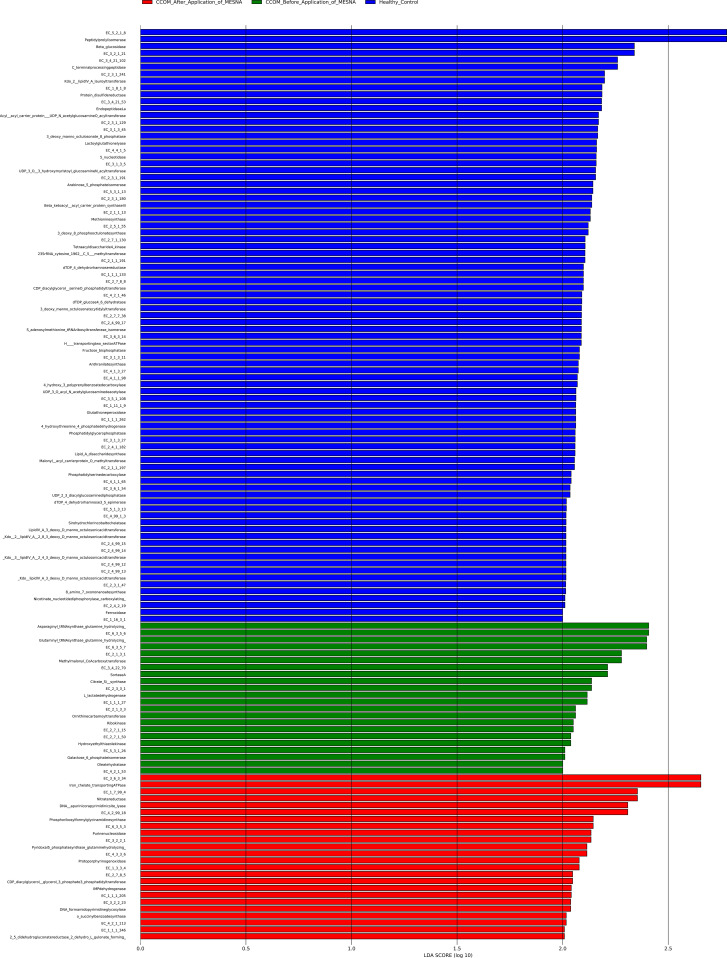
Functional enrichment analysis using linear discriminant analysis effect size (LEfSe) of enzyme commission (EC) numbers. Healthy controls (blue) show enrichment in biosynthetic pathways essential for mucosal homeostasis: lipopolysaccharide biosynthesis, phospholipid metabolism, and oxidative stress defense. CCOM Before MESNA (green) exhibits enrichment in resource acquisition pathways: iron-chelate transport, nitrate reductase (anaerobic metabolism), and amino acid biosynthesis. CCOM After MESNA (red) shows transitional metabolic state with minimal discriminative features, including DNA repair enzymes and partial restoration of membrane biosynthesis. LDA score > 2.0, Kruskal-Wallis test α = 0.05. All functional features represent PICRUSt2-predicted metabolic potential inferred from 16S rRNA taxonomic composition and should be interpreted as hypothesis-generating rather than direct measurements of enzymatic activity.

Functional inference (**Figure 8**) predicts distinct metabolic profiles, though experimental validation is required. Healthy controls exhibited enrichment in multiple biosynthetic pathways essential for maintaining mucosal homeostasis. The most prominent feature was lipopolysaccharide biosynthesis, e.g., the complete cascade of Kdo (3-deoxy-D-manno-octulosonate) transferases (EC 2.4.99.12, EC 2.4.99.13, EC 2.4.99.14, EC 2.4.99.15), lipid A disaccharide synthase (EC 2.4.1.182), and supporting enzymes for precursor synthesis (EC 2.7.7.38, EC 2.5.1.55, EC 3.1.3.45).

Phospholipid metabolism was equally prominent, with key enzymes for membrane backbone synthesis (CDP-diacylglycerol--serine O-phosphatidyltransferase EC 2.7.8.8), phospholipid maturation (phosphatidylserine decarboxylase EC 4.1.1.65, phosphatidylglycerophosphatase EC 3.1.3.27), and fatty acid modification (malonyl-[acyl-carrier protein] O-methyltransferase EC 2.1.1.197). This membrane-centric biosynthetic profile is consistent with a community dominated by Gram-negative commensal bacteria capable of forming stable protective biofilms in healthy middle ear tissue. Additional enriched pathways included cofactor synthesis (nicotinate nucleotide diphosphorylase EC 2.4.2.19 for NAD^+^), oxidative stress defense (glutathione peroxidase EC 1.11.1.9, protein-disulfide reductase EC 1.8.1.8), and central carbon metabolism (fructose-bisphosphatase EC 3.1.3.11).

In contrast, CCOM samples before MESNA application exhibited predicted enrichment in resource acquisition and competition-associated pathways. Iron acquisition was prominently represented by iron-chelate transporting ATPase (EC 3.6.3.34), which facilitates ferric-siderophore import across the inner membrane—a key virulence-associated mechanism for pathogenic bacteria competing for iron in host tissues. Anaerobic metabolism was indicated by nitrate reductase (EC 1.7.99.4), enabling nitrate respiration under oxygen-limited conditions typical of biofilm microenvironments.

Inferred central carbon metabolism enzymes included L-lactate dehydrogenase (EC 1.1.1.27) for fermentative growth and citrate (Si)-synthase (EC 2.3.3.1) for TCA cycle function. Amino acid biosynthesis was represented by ornithine carbamoyl transferase (EC 2.1.3.3), a key enzyme in arginine metabolism. Supporting metabolism included ribokinase (EC 2.7.1.15) for ribose phosphorylation, hydroxyethylthiazole kinase (EC 2.7.1.50) for thiamine biosynthesis, and galactose-6-phosphate isomerase (EC 5.3.1.26) for carbohydrate utilization. Specialized enzymes included oleate hydratase (EC 4.2.1.53) for lipid modification.

Following MESNA application, the functional profile exhibited minimal discriminative features. The few enriched enzymes included protoporphyrinogen oxidase (EC 1.3.3.4) for heme biosynthesis, pyridoxal 5’-phosphate synthase (EC 4.3.3.6) for vitamin B6 production, and DNA repair enzymes (DNA formamidopyrimidine glycosylase EC 3.2.2.23, DNA-(apurinic or apyrimidinic site) lyase EC 4.2.99.18). These findings suggest a response to MESNA-induced oxidative damage, alongside incipient attempts to restore cellular growth.

Nucleotide metabolism was represented by IMP dehydrogenase (EC 1.1.1.205), purine nucleosidase (EC 3.2.2.1), and phosphoribosylformylglycinamidine synthase (EC 6.3.5.3). CDP-diacylglycerol--glycerol-3-phosphate 3-phosphatidyltransferase (EC 2.7.8.5), o-succinyl benzoate synthase (EC 4.2.1.113) for menaquinone synthesis, and 2,5-didehydrogluconate reductase (EC 1.1.1.346) were observed as well.

### 3.7 Differential abundance analysis

Compositional differential abundance testing (**Table 1**) was performed using ALDEx2 with CLR transformation. Monte Carlo sampling was used to model technical variation by drawing from a Dirichlet distribution. Kruskal-Wallis tests were applied across the three clinical groups (Healthy Controls, CCOM Before MESNA, CCOM After MESNA) to evaluate robust global shifts in individual taxon abundances.

**Table 1:** Compositional differential abundance testing using ALDEx2 with CLR transformation and Kruskal-Wallis tests across the three clinical study groups.

Genus	KW *p*-value	KW *q*-value FDR	GLM *p*-value	GLM *q*-value FDR
*Prevotella*	0.0047*	0.2359	0.0019*	0.0245*
*Pseudomonas*	0.0284*	0.3394	7e-04*	0.0205*
*Pseudomonas_*E_647464	0.0295*	0.3192	0.0125*	0.1249
*Cutibacterium*	0.0317*	0.3886	0.0483*	0.3072
*Leptotrichia*	0.0351*	0.3576	0.0042*	0.0601
*Pelomonas*	0.0409*	0.3416	0.0142*	0.1283
*Segatella*	0.0498*	0.3922	0.01*	0.0863
*Neisseria*	0.0553	0.4041	0.0059*	0.064
*Lawsonella*	0.0879	0.4299	0.0547	0.2669
*Listeria*	0.1198	0.4875	0.008*	0.0969
*Streptobacillus*	0.1377	0.5155	0.0362	0.1869
*Brevundimonas*	0.1381	0.5097	0.0378*	0.1937
*Porphyromonas*	0.146	0.5312	0.0126*	0.1165
*Veillonella*	0.1478	0.5317	0.0329*	0.1856
*Fusobacterium*	0.16	0.5417	0.0084*	0.0895
*Bacteroides*	0.1769	0.567	0.0742*	0.2793
*Proteus*	0.1878	0.5388	0.1718	0.4545
*Peptoniphilus_*A	0.1921	0.5501	0.1598	0.4577
*Porphyromonas_*A_859426	0.1948	0.5522	0.1405	0.4189
*Mycolicibacterium*	0.225	0.5967	0.0355*	0.1896

KW *p*-value: Kruskal-Wallis *p*-value; KW Q-value FDR: Kruskal-Wallis Q-value with FDR correction; GLM *p*-value, Generalized Linear Mode *p*-value; GLM Q-value FDR, Generalized Linear Model Q-value with FDR correction; *: *p*-value < 0.05.

While a subset of genera exhibited nominal statistical differences prior to multiple testing correction, only *Pseudomonas* (GLM q = 0.0205) and *Prevotella* (GLM q = 0.0245) achieved significance after Benjamini-Hochberg FDR correction in the GLM framework. Under the non-parametric Kruskal-Wallis test, however, neither genus survived FDR correction (*Pseudomonas* KW q = 0.3394; *Prevotella* KW q = 0.2359), and all remaining genera fell substantially above the significance threshold under both frameworks. Notably, the two genera achieving significance under ALDEx2 GLM-FDR correction (*Pseudomonas* and *Prevotella*) were concordant with LEfSe candidate signatures, providing internal validation for the exploratory analysis.

This divergence likely reflects differences in underlying statistical assumptions: the GLM captures linear compositional trends and benefits from the variance-stabilizing CLR transformation, while the non-parametric Kruskal-Wallis test is more conservative under conditions of high inter-individual variability. Together, both approaches highlight the marked compositional heterogeneity of the dysbiotic state, and the significant taxa identified here should be interpreted in conjunction with the rank-based LEfSe biomarkers described in Section 3.4.

#### 3.7.1 Paired analysis confirms stochastic recolonization dynamics

To strictly evaluate the within-patient shifts induced by the MESNA application, we performed an exclusive paired differential abundance analysis. We utilized the paired Student’s t-test module within ALDEx2 (paired.test = TRUE), matching the exact ‘Before’ and ‘After’ samples for each of the 13 patients.

Consistent with the multi-directional trajectories observed in our paired beta-diversity analyses (Robust Aitchison PCoA), no single genus maintained statistical significance across the cohort following strict Benjamini-Hochberg FDR correction (all adjusted q > 0.05). Furthermore, standardized effect sizes remained small across all taxa.

The absence of universally conserved taxonomic biomarkers following FDR correction is consistent with the high inter-individual stochasticity observed in the paired beta-diversity analysis and provides quantitative support for a priority-effects model of post-MESNA recolonization.

### 3.8 Random forest classification and Boruta feature selection

A Random Forest classifier trained on genus-level relative abundances achieved an out-of-bag (OOB) accuracy = 0.73 and a five-fold cross-validated (CV) accuracy = 0.76, with a multiclass Area Under the Curve (AUC) = 0.964, indicating preliminary discriminatory capacity among the three clinical groups (**Table 2**), pending validation in larger cohorts.

**Table 2:** Feature importance of discriminatory genera identified by Boruta-random forest classification.

Feature	Importance
*Cutibacterium*	10.2181632999324
*Leptotrichia*	10.9006071328138
*Listeria*	8.47441685523573
*Neisseria*	12.837832958193
*Pseudomonas*	11.4219296894463
*Segatella*	10.4530304440226

Out-Of-Bag (OOB) accuracy, 0.73; Cross-Validation (CV) accuracy, 0.76; Area Under the Curve (AUC), 0.964.

Boruta feature selection identified six discriminatory genera, which were subsequently used to train the Random Forest classifier. Feature importance, expressed as Mean Decrease in Accuracy, ranked *Neisseria* as the most discriminatory taxon (MDA = 12.84), followed by *Pseudomonas* (11.42), *Leptotrichia* (10.90), *Segatella* (10.45), *Cutibacterium* (10.22), and *Listeria* (8.47).

Given the limited sample size, these performance metrics should be interpreted cautiously. Despite five-fold cross-validation and out-of-bag error estimation, models trained on small cohorts remain susceptible to overfitting, and external validation in an independent dataset will be required to confirm the generalizability of these discriminatory features.

## 4 Discussion

The present study integrates taxonomic profiling, compositional network inference, and predicted functional analysis to characterize the microbial ecology of pediatric cholesteatomatous chronic otitis media and the ecological response to MESNA-assisted dissection. Four principal findings emerge from this multimodal analysis. First, CCOM is associated with a profound reduction of microbial network connectivity — from 185 edges in healthy controls to two edges in CCOM Before MESNA — representing a fundamental loss of competitive exclusion and ecological resilience that defines the dysbiotic state. Second, *Cutibacterium* transitions from an integrated community hub in health to an ecologically isolated candidate pathobiont in disease, remaining network-excluded following MESNA application in a pattern consistent with biofilm-mediated persistence. Third, MESNA acts as a non-specific ecological press disturbance that disrupts the disease-associated community but fails to restore a healthy compositional state, as confirmed by the stochastic, multi-directional within-patient trajectories revealed by the paired analysis. Fourth, the post-treatment community exhibits early-stage successional dynamics characterized by high inter-individual stochasticity and pioneer recolonization rather than deterministic recovery. The following sections discuss each of these findings in the context of microbial ecology theory and their clinical implications, with the explicit caveat that all mechanistic interpretations are hypothesis-generating and require experimental validation through metatranscriptomics, *in vitro* co-culture, or targeted gene expression assays.

### 4.1 Network fragmentation predicts functional collapse

Our most striking finding is the quantitative demonstration of network collapse in CCOM. The reduction from 185 microbial interactions in healthy controls to two edges with four isolated genera in CCOM Before MESNA patients represents loss of ecological connectivity. This is a fundamental disruption of the ability of the microbiota to self-regulate through competitive exclusion, resource partitioning, and antagonistic interactions (Proulx et al., 2005; Burton et al., 2022; Jacob and Reguera, 2022). In ecological theory, network complexity and functional redundancy are primary determinants of ecosystem resilience (Bascompte and Jordano, 2007): when perturbations occur, highly connected communities can compensate through alternative pathways and species substitutions, whereas simplified communities lack this buffering capacity and are prone to regime shifts toward pathological states (Scheffer et al., 2001; Wang et al., 2024).

It should be noted, however, that the dramatic reduction in network edges, while ecologically interpretable, may also partly reflect the reduced statistical power of co-occurrence inference at small sample sizes. The conservative consensus approach, which requires concordance across three independent algorithms, prioritizes specificity over sensitivity and may underestimate true ecological connectivity. These findings should therefore be considered hypothesis-generating, pending replication in larger cohorts.

Critically, this network simplification also correlated with predicted functional shifts. In healthy controls, the dense interaction network was associated with enrichment of biosynthetic pathways characteristic of metabolically diverse, actively growing communities (Karp et al., 2019; Kanehisa et al., 2023). These pathways are energetically expensive and require stable nutrient availability typically found in homeostatic environments. It is important to note that these findings reflect predicted metabolic potential rather than demonstrated reprogramming and should be considered hypothesis-generating pending metatranscriptomic validation.

With this caveat in mind, the CCOM Before MESNA network collapse to two isolated dyadic interactions correlated with predicted functional streamlining toward resource competition and anaerobic adaptation. This functional-structural coupling suggests a correlation between network architecture and predicted metabolic potential, consistent with a scenario where loss of competitive antagonism permits pathobiont expansion and would select for competitive strategies over biosynthetic investment.

### 4.2 Taxonomic homogenization reflects stochastic succession

In the genus-level LEfSe analysis, healthy controls exhibited 42 discriminative genera, spanning diverse functional guilds including obligate anaerobes (*Fusobacterium, Veillonella*), facultative anaerobes (*Haemophilus*), and aerobic environmental taxa. This diversity underscores the metabolic versatility required to maintain homeostasis across varying oxygen tensions and immune pressures (Proulx et al., 2005).

The detection of *Fusobacterium* and *Listeria* in healthy controls warrants contextual discussion, as both genera are classically associated with pathogenic presentations in clinical settings.

Regarding *Fusobacterium*, growing evidence supports a commensal—and even protective role—in the pediatric upper respiratory tract microbiota. Sokolovs-Karijs et al. (2024) identified *Fusobacterium* among the five most abundant genera in adenoids of healthy children (Sokolovs-Karijs et al., 2024), while Jo et al. (2025) specifically designated *Fusobacterium nucleatum* as an indicator species of healthy adenoid microbiota in children aged 6–12 years, with its abundance decreasing in chronic otitis media with effusion—a finding that directly parallels our observation of its dominance in healthy controls rather than disease groups (Jo et al., 2025). Santos-Cortez et al. (2016) additionally detected elevated *Fusobacterium* in chronic otitis media middle ear samples, suggesting a dynamic contextual role across disease states (Santos-Cortez et al., 2016). Collectively, these findings support the interpretation that Fusobacterium occupies a commensal niche in pediatric upper airway and middle ear tissue, with pathogenic potential emerging only under specific dysbiotic conditions.

The presence of *Listeria* in healthy middle ear tissue is less precedented in the literature and must be interpreted cautiously. Clinical listeriosis is predominantly associated with immunocompromised individuals and neonates; however, environmental and non-pathogenic Listeria species, e.g., *L. innocua*, *L. seeligeri*, are ubiquitously distributed across soil, food matrices, and mucosal surfaces (Orsi and Wiedmann, 2016).

Detection of *Listeria* by 16S rRNA amplicon sequencing in respiratory and gastrointestinal microbiome studies of healthy subjects has been previously reported without associated pathology, likely reflecting the presence of environmental phylotypes rather than clinically relevant strains (Grif et al., 2003; Hafner et al., 2021). Critically, *Listeria* did not achieve statistical significance in any inter-group comparison in our dataset, and its independent identification as a discriminatory taxon by the Boruta-Random Forest pipeline supports a context-dependent community role rather than an incidental contaminant. Given that 16S rRNA sequencing does not resolve species-level identity, future studies incorporating whole-genome sequencing would be required to confirm whether the detected phylotypes correspond to pathogenic or environmental lineages.

Furthermore, although negative extraction controls confirmed the absence of reagent-associated contamination, cross-sample contamination during tissue processing cannot be formally excluded given the clinical laboratory setting. However, the consistent detection of *Listeria* across independent samples, its community-level behavior as a discriminatory taxon in the Boruta-Random Forest pipeline, and its absence of statistical significance in inter-group comparisons collectively suggest that its detection is unlikely to reflect systematic contamination alone, though its ecological significance remains to be determined.

The CCOM Before MESNA microbiota showed a reduction to only two discriminative genera (*Pseudomonas* and *Pelomonas*), quantifying the taxonomic simplification accompanying network collapse. While *Staphylococcus* and *Cutibacterium* dominated the composition data, their high inter-individual variability prevented statistical significance in LEfSe. Conversely, *Pseudomonas* and *Pelomonas* likely represent consistently present low-abundance taxa that increase predictably in dysbiosis, functioning as reliable biomarkers rather than primary drivers (Han and Vaishnava, 2023).

This taxonomic homogenization suggests that CCOM Before/After MESNA states represent early seral stages of ecological succession. In ecological theory, pioneer stages colonizing disturbed habitats are characterized by high stochasticity, where priority effects allow whichever colonizer arrives first to dominate, leading to high inter-site variability (Wang et al., 2024).

Similarly, CCOM After MESNA exhibited only two discriminative genera (*Gemella* and *Brevundimonas*), indicating a state where stochastic colonization overwhelms deterministic assembly. Full recovery would require extended recolonization time for deterministic processes—such as competitive exclusion and environmental filtering—to converge the community toward the sTable 42-genus signature observed in healthy controls (Jacob and Reguera, 2022; Wang et al., 2024).

### 4.3 Compositional displacement reveals incomplete microbial restoration

The PCoA analysis reinforces this interpretation. The spatial segregation of healthy controls from clinical samples along PC1 reflects fundamental ecological state differences. The Aitchison distance metric, utilized through CLR transformation (Anderson, 2017), effectively highlights these shifts in relative abundances.

CCOM Before MESNA samples clustered tightly in a log-ratio space defined by the *Staphylococcus-Cutibacterium* axis, indicating a pathological attractor state (Scheffer et al., 2001). This represents a stable but unhealthy equilibrium from which the microbiota cannot spontaneously escape. The divergent trajectory of CCOM After MESNA samples away from this attractor suggests that the intervention disrupts this equilibrium, creating the necessary conditions for potential ecological recovery.

Critically, the non-significant paired PERMANOVA result is a positive confirmation that MESNA does not induce a stereotyped, uniform community transition but instead releases each patient’s community from the dysbiotic attractor into an individualized successional trajectory. This is ecologically consistent with a priority-effects model and clinically consistent with the known inter-patient variability in CCOM recurrence outcomes.

### 4.4 *Cutibacterium acnes* as context-dependent pathobiont

*Cutibacterium acnes* is frequently dismissed from metagenomic analyses as a culture contaminant in clinical microbiology due to its ubiquity on human skin (Portillo et al., 2013). However, multiple lines of evidence argue against artifactual contamination in our dataset: 1) high prevalence across independent samples; 2) active network integration in healthy controls, forming competitive and cooperative interactions impossible for external contaminants; 3) context-dependent abundance shifts correlating with disease state; and 4) RNA-based detection indicating metabolic activity. While *C. acnes* colonizes multiple anatomical sites with niche-specific roles (Achermann et al., 2014)—skin, prosthetic joints, respiratory tract—its ecological behavior is highly context-dependent.

#### 4.4.1 Competitive and cooperative roles constrain pathogenic potential

*Cutibacterium acnes* exemplifies context-dependent pathogenicity. In healthy controls, it is a highly integrated community member, participating in a robust 185-edge network where its growth is constrained by competitive and cooperative interactions. This state is characterized by balanced metabolism, phospholipid synthesis, and oxidative stress defense, suggesting that *C. acnes* may contribute to mucosal homeostasis through lipid and short-chain fatty acid (SCFA) generation (Bortz et al., 1990; Nakase et al., 2022).

In CCOM Before MESNA dysbiosis, despite a 92.3% prevalence, *C. acnes* undergoes a profound behavioral shift toward ecological isolation, evidenced by its complete absence in co-occurrence networks. However, its absence in the inferred networks should be cautiously interpreted: our conservative consensus approach may fail to detect weak but biologically relevant associations in which *C. acnes* participates. As competitive antagonism collapses, *C. acnes* ceases mutualistic partnerships to independently exploit the cholesteatoma’s unique, resource-limited niche—rich in lipids but depleted in oxygen and iron (Bortz et al., 1990; Bell et al., 2016).

#### 4.4.2 Metabolic reprogramming in dysbiosis

Indirect evidence of *C. acnes* mutualism persists through the positive co-occurrence of its metabolic partners, such as *Lawsonella*, *Corynebacterium*, and *Peptoniphilus*_A. A cryptic syntrophy likely occurs where *Lawsonella* ferments carbohydrates into SCFAs (Reigstad and Kashyap, 2013), which *C*. *acnes* then consumes while catabolizing lipids (Bortz et al., 1990). This is supported by LEfSe results showing the simultaneous enrichment of carbohydrate metabolism and lipid modification pathways, indicating specialized niche partitioning between these taxa.

While 16S rRNA sequencing cannot resolve the six *C. acnes* phylotypes (Fitz-Gibbon et al., 2013), functional predictions suggest potential enrichment of pathogenic lineages in CCOM Before MESNA. Functional prediction analysis suggested potential enrichment of pathobiont- associated gene families (iron acquisition, biofilm formation, lipid catabolism) in CCOM Before MESNA samples (Fujikawa et al., 2022); however, these represent computational inferences that require experimental validation, as 16S rRNA sequencing does not directly measure gene expression.

#### 4.4.3 Biofilm persistence drives risk for recurrence

Remarkably, *C. acnes* remains network-excluded in CCOM After MESNA. This persistent ecological isolation suggests that standard antimicrobial activity is insufficient to eradicate biofilm reservoirs or intracellular populations. This latent state may drive high recurrence rates; the bacterium remains poised for re-expansion if dysbiotic conditions return. Based on these ecological observations, future therapeutic strategies could consider physical removal to include quorum sensing inhibitors to target persistent biofilms, and probiotic introduction of antagonistic commensals to force network reintegration and restore colonization resistance (Burton et al., 2022; Jacob and Reguera, 2022).

However, these interpretations assume strain-level differences may drive the observed ecological shifts, though our 16S approach cannot distinguish phylotypes. Validation through shotgun metagenomics or strain isolation is required.

### 4.5 Staphylococcal dominance: defense or dysfunction?

#### 4.5.1 Intrageneric competition limits virulence but not disease

The dominance of *Staphylococcus* in CCOM Before MESNA samples is ecologically significant. While *S. aureus* is traditionally associated with aggressive bone resorption and osteoclast activation in cholesteatomas (Akoua-Koffi et al., 2022; Frank et al., 2022), the predominance of coagulase-negative staphylococci (CoNS)—such as *S. capitis, S. hominis*, and *S. saccharolyticus*—suggests a process of intrageneric competitive exclusion. CoNS produce interference factors, including serine protease inhibitors and antimicrobial peptides, which specifically inhibit *S. aureus* colonization (Frank et al., 2022). This represents a form of colonization resistance, where resident commensals prevent the establishment of more virulent species.

Despite this resistance, CoNS-mediated defense is insufficient to restore homeostasis. The persistent network collapse and the functional shift toward iron competition indicate that CoNS dominance constitutes only a partial defense. While it may prevent the accelerated bone destruction associated with *S. aureus*, it fails to recover the complex, 185-edge interactome of healthy tissue. The presence of species like *S. schweitzeri*—which possesses virulence genes homologous to *S. aureus* (Stewart and Franklin, 2008)—further suggests that pathogenic potential is determined by strain-level variation within the complex.

#### 4.5.2 Biofilm architecture creates hypoxic microenvironments

Functionally, the enrichment of central carbon metabolism and nitrate reductase is consistent with predicted adaptation to the stratified microenvironments of biofilms. Biofilms generate steep oxygen gradients, with aerobic respiration limited to the interface and hypoxia prevailing in deeper layers (Heaf et al., 1983). Nitrate reductase allows facultative anaerobes like *Staphylococcus* to utilize nitrate as a terminal electron acceptor, supporting growth in hypoxic depths where obligate aerobes perish.

This metabolic versatility positions CoNS as a candidate keystone pathobionts. Rather than driving primary tissue destruction, they enable dysbiotic persistence by modifying the environment—through biofilm formation and oxygen depletion—in ways that favor the expansion of anaerobic pathobionts (Rosenblatt et al., 2017; Frank et al., 2022).

### 4.6 MESNA as an ecological press disturbance

#### 4.6.1 Pioneer species dominate early successional stages

Beyond its mucolytic role, MESNA acts as an ecological press disturbance, inducing taxonomic homogenization rather than a return to a healthy state. The absence of LEfSe-discriminative genera and high inter-individual variability—where dominance shifts inconsistently between taxa like *Corynebacterium* and *Pseudomonas*—suggests a continuous selection pressure favoring stress-tolerant generalists. The observed decline in Shannon diversity and Chao1 richness post-treatment is consistent with early-stage secondary succession, where pioneer species (*Corynebacterium*, *Eubacterium*_B) rapidly colonize the disturbed niche before competitive interactions can re-stabilize the community (Wang et al., 2024).

The most significant ecological result is the reemergence of competitive exclusion in CCOM After MESNA networks. This transition from purely cooperative, mutualism-dominated dysbiosis toward mixed competitive-cooperative dynamics indicates the microbiota is beginning to partition niches—a hallmark of community stabilization (Jacob and Reguera, 2022; Wang et al., 2024). However, PCoA analysis confirms that while the microbiota has escaped the pathological attractor (Scheffer et al., 2001), it remains spatially segregated from healthy controls, indicating incomplete successional recovery.

#### 4.6.2 Oxidative stress responses indicate biofilm disruption

Functionally, the loss of iron acquisition and anaerobic signatures suggests that MESNA disrupts the competitive landscape by dismantling biofilms. The concomitant enrichment of DNA repair enzymes reflects adaptation to oxidative stress induced by MESNA’s thiol chemistry (Reigstad and Kashyap, 2013). While a shift toward membrane biosynthesis indicates that pioneer recolonizers are transitioning from stress response to active growth (Karp et al., 2019; Kanehisa et al., 2023), the persistence of *C. acnes* and the expansion of *Pseudomonas* temper this optimism.

#### 4.6.3 Clinical implications for targeted antibiotic prophylaxis

*Pseudomonas* prevalence increased from four to seven patients post-treatment. This expansion, despite MESNA’s documented antimicrobial activity (Rosenblatt et al., 2017; Miranda et al., 2022), suggests strain-specific resistance, rapid recolonization, or quorum sensing-mediated resilience (Qin et al., 2022; Modi and Kiverniti, 2024). This finding has direct clinical implications: post-surgical antibiotic prophylaxis targeting *Pseudomonas* may be essential to prevent biofilm re-establishment and subsequent cholesteatoma recurrence (Akoua-Koffi et al., 2022; Modi and Kiverniti, 2024).

### 4.7 Pioneer recolonizers and ecological facilitation in postMESNA succession

The emergence of saprophytic bacteria in CCOM After MESNA suggests its role as pioneer recolonizers rather than *de novo* pathogens. As decomposers, these bacteria likely metabolize residual keratin and disrupted biofilms, releasing nutrients that facilitate colonization by later-stage commensals (Li et al., 2018; Lv et al., 2022). This is particularly relevant for non-*S. pneumoniae* streptococci—keystone commensals that maintain homeostasis through syntrophic biofilm formation and pathogen antagonism (Jacob and Reguera, 2022).

This process represents ecological facilitation, where pioneer species modify the disturbed environment to enable the establishment of complex communities (Wang et al., 2024). The positive co-occurrence between *Paracoccus* and *Streptococcus* in post-treatment networks supports this transition toward cooperative colonization. However, the potential for saprophytic-induced chronic granulomatous inflammation cannot be ignored (Fitz-Gibbon et al., 2013); longitudinal monitoring is essential to distinguish transient successional colonization from persistent pathogenic potential requiring intervention.

### 4.8 Translating ecology into clinical practice

Our findings support shifting from traditional pathogen elimination toward restoring ecological resilience through network reconstruction.

#### 4.8.1 Network metrics as clinical biomarkers

Quantitative tracking of network connectivity (185 → 2 → 3 edges) can complement traditional diagnostics. Patients with minimal post-surgical connectivity may require extended antimicrobial prophylaxis or probiotic intervention.

#### 4.8.2 Anti-biofilm strategies beyond mucolysis

Since *C. acnes* and *Pseudomonas* persist in CCOM After MESNA, combining the mucolytic with DNase I (matrix targeting), furanones (quorum sensing), or iron chelators could enhance pathobiont clearance (Heaf et al., 1983; Qin et al., 2022).

#### 4.8.3 Probiotic-mediated competitive exclusion

Topical “seeding” of the post-surgical niche with antagonistic commensals (e.g., specific CoNS strains, *Corynebacterium* spp., or streptococci) could accelerate recovery by forcing network reintegration and inhibiting pathobiont expansion (Burton et al., 2022; Jacob and Reguera, 2022).

#### 4.8.4 Therapeutic repurposing of MESNA

MESNA’s antimicrobial effects suggest its utility could extend to other biofilm-associated conditions, e.g., chronic wounds, device-associated infections, or cystic fibrosis (Rosenblatt et al., 2017; Miranda et al., 2022).

### 4.9 Study constraints and future trajectories

Several limitations warrant discussion. First, the decision to focus our RNA-based workflow to exclusively detect metabolically active microbiota. While a direct metatranscriptomic profiling of mRNA would enable measurement of active gene expression, this strategy was beyond the scope of this study for two reasons. On one hand, the primary objective of rRNA-derived cDNA amplification was enhanced detection sensitivity for the metabolically active community in a low-biomass, high-host-background sample type, rather than functional gene expression profiling *per se*. On the other hand, low-biomass middle ear tissue biopsies yield insufficient total RNA for mRNA-selective capture after ribosomal RNA depletion. The resulting workflow is functionally analogous to standard 16S amplicon sequencing at the community composition level, with the additional advantage of preferentially detecting metabolically active organisms.

Second, the genus-level resolution inherent to 16S rRNA V3–V4 amplicon sequencing is a particularly relevant constraint for several taxa central to CCOM pathogenesis identified in this study. Within *Staphylococcus*, coagulase-negative species and *S. aureus* carry markedly divergent virulence profiles — a distinction with direct clinical implications for bone resorption and disease severity that cannot be resolved at the genus level. Within *Cutibacterium*, the six recognized phylotypes of *C. acnes* differ substantially in their association with inflammatory disease, biofilm formation capacity, and metabolic repertoire (Fitz-Gibbon et al., 2013); our 16S-based ecological observations regarding *C. acnes* therefore represent phylotype-agnostic inferences that require strain-level resolution for mechanistic validation. Future studies incorporating whole-genome shotgun metagenomics or targeted amplicon sequencing of phylotype-discriminatory *loci* will be essential to determine whether the ecological transitions described here are driven by shifts in community membership, shifts in phylotype composition within persistent genera, or both.

Third, a structural limitation of the present study concerns the heterogeneous sampling design across clinical groups. CCOM Before and After MESNA samples were collected from the same thirteen pediatric patients in a paired longitudinal fashion, enabling direct within-individual assessment of MESNA’s microbiological effect while controlling for inter-individual biological variability. In contrast, Healthy Control samples were obtained from an independent cross-sectional cohort of eleven subjects, precluding matched comparisons between disease and health states.

A further consideration concerns statistical power. The present cohort—thirteen paired CCOM patients and eleven independent healthy controls—is modest by microbiome study standards. While formal power analysis for high-dimensional compositional data remains methodologically complex, these sample sizes are consistent with published exploratory studies of cholesteatoma-associated microbiota (Frank et al., 2022; Fujikawa et al., 2022), a low-prevalence condition where recruitment is limited by surgical frequency. The paired design was specifically selected to maximize statistical efficiency by eliminating inter-individual biological variability, partially compensating for the limited sample size. Notably, the results indicate that MESNA explains only 3.2% of within-patient compositional variance. This suggests that even in larger cohorts, the effect size would remain modest—a finding that is biologically informative rather than a mere power limitation, as it supports the priority-effects model of post-MESNA recolonization. Nevertheless, the reduced power for detecting weak compositional differences—particularly in differential abundance testing, where only two genera survived FDR correction—must be considered when interpreting these findings. External validation in larger, prospectively designed cohorts remains a priority.

This design heterogeneity has two principal statistical consequences. On one hand, the paired structure of the CCOM samples was not exploited in the primary statistical framework: PERMANOVA with Aitchison distances and ALDEx2 differential abundance testing were applied treating all three groups as independent, which is conservative but underutilizes the statistical power conferred by pairing. To address this limitation, a paired PERMANOVA was implemented using patient identity as a blocking factor (strata argument in adonis2), and paired differential abundance testing was conducted using ALDEx2 with paired.test = TRUE.

On the other hand, between-group comparisons involving Healthy Controls—particularly the HC vs. CCOM Before MESNA contrast—should be interpreted as cross-sectional associations rather than causal trajectories, as the HC cohort remains independent and cross-sectional. Unmodelled confounders, including age distribution differences, antibiotic exposure history, and anatomical site-specific variation between healthy and cholesteatomatous tissue, may contribute to the observed compositional differences independently of disease status. While the magnitude of the observed shifts suggests these findings are robust to moderate confounding, formal causal inference would require a prospective matched-control design with covariate adjustment.

These limitations do not invalidate the primary findings of this study, which are framed as hypothesis-generating and exploratory. However, they underscore the need for replication in larger, prospectively designed cohorts where paired sampling extends to healthy controls, enabling the application of longitudinal mixed-effects models and the formal assessment of ecological recovery trajectories across post-surgical timepoints.

Fourth, while PICRUSt2 provides valuable insights into metabolic potential by inferring functional gene content from 16S rRNA sequences, it predicts genomic capacity rather than measuring active gene expression (Douglas et al., 2020). The functional shifts we report—including enrichment of iron acquisition systems, lipid catabolic enzymes, and DNA repair pathways—represent predicted metabolic potential based on the taxonomic composition of each sample. Actual metabolic activity can only be confirmed through direct measurement of gene expression, protein abundance, or metabolite profiles. Future studies incorporating these approaches, combined with targeted RT-qPCR validation of key pathways (e.g., iron acquisition genes in CCOM Before MESNA, lipid biosynthesis in healthy controls), would strengthen the mechanistic understanding of CCOM pathogenesis and inform more targeted therapeutic interventions.

Fifth, our single post-intervention sampling point prevents the assessment of longitudinal recovery; serial sampling is essential to distinguish transient recolonizers from persistent pathobionts and to identify critical windows for clinical intervention.

Sixth, statistical co-occurrence networks infer associations rather than mechanistic interactions. Network inference may underestimate true connectivity due to limited statistical power for detecting weak-to-moderate correlations. The consensus approach (intersection of three algorithms) prioritizes specificity over sensitivity, potentially excluding biologically relevant interactions. Validation in larger cohorts is essential to confirm the extent of network collapse observed here. Moreover, *in vitro* co-culture experiments with representative isolates are needed to directly test metabolic cross-feeding, competitive inhibition, or spatial co-localization (Burton et al., 2022).

Finally, detailed clinical and demographic metadata (i.e., patient age distribution, sex, disease severity staging, prior antibiotic exposure history) were not systematically collected as part of the primary study design and are therefore unavailable for formal covariate adjustment. This represents a genuine limitation for assessing potential confounding effects on microbiota composition. We note that the paired sampling design inherently controls for fixed inter-individual confounders, including baseline host characteristics, partially mitigating the absence of demographic covariate adjustment for the within-patient MESNA comparison. However, between-group comparisons involving the independent healthy control cohort remain susceptible to unmodelled demographic confounding. Future prospective studies should incorporate standardized clinical metadata collection, e.g., including antibiotic exposure within 3–6 months prior to sampling, disease staging by established criteria, complete demographic characterization, to enable formal assessment of these variables and improve comparability across study populations.

Finally, integrating host factors—including immune profiles, inflammatory cytokines, and genetic polymorphisms in pattern recognition receptors—will be critical to elucidating the complex microbe-host-environment interactions driving dysbiosis and ecological recovery in CCOM (Lv et al., 2022).

## 5 Conclusions

Our study identifies network collapse as an ecological signature of dysbiosis in pediatric cholesteatomatous chronic otitis media (CCOM). The transition from a stable, high-connectivity healthy state to the fragmented interactome seen in disease reflects a fundamental loss of competitive exclusion and ecological resilience. While MESNA-assisted dissection disrupts this pathological equilibrium and initiates secondary succession, the resulting taxonomic turnover and functional simplification indicate that successional recovery remains incomplete.

*Cutibacterium acnes* emerges as a model of context-dependent pathogenicity, transitioning from an integrated community member in health to an ecologically isolated candidate pathobiont in disease, and finally to a persistent but network-excluded colonizer post-treatment. This ecological plasticity and metabolic versatility position *C. acnes* as an ecologically isolated candidate pathobiont whose survival strategies are central to disease persistence. Our multimodal integration of taxonomic, network, and functional data confirms that CCOM is defined by a profound restructuring of ecological architecture and metabolic reprogramming.

These findings advocate for a paradigm shift from pathogen eradication to ecological restoration. Future interventions should prioritize network reconstruction—through targeted anti-biofilm strategies, probiotic seeding, and the facilitation of competitive exclusion—rather than relying solely on antimicrobials. Merging microbial ecology with clinical microbiology provides a robust frontier for improving long-term outcomes in CCOM and other chronic-recurrent diseases.

While our functional predictions require experimental validation through metatranscriptomics or targeted gene expression analysis, the strong correlation between network structure, taxonomic composition, and predicted metabolic potential provides a robust foundation for hypothesis-driven mechanistic studies and the development of ecology-based therapeutic strategies in pediatric CCOM.

## Data Availability

The sequencing data generated in this study have been deposited in the European Nucleotide Archive (ENA) at EMBL-EBI under accession number [PRJEB108671] (https://www.ebi.ac.uk/ena/browser/view/PRJEB108671).
